# When care turns to harm: Unveiling abusive behaviors of older towards their caregivers

**DOI:** 10.1192/j.eurpsy.2025.1766

**Published:** 2025-08-26

**Authors:** S. von Humboldt, N. Ilyas, I. Leal

**Affiliations:** 1 William James Center for Research, ISPA – Instituto Universitário, Lisbon, Portugal; 2 Center for Clinical Psychology, University of the Punjab, Lahore, Pakistan

## Abstract

**Introduction:**

Violent, abusive, and harmful behavior enacted by older adults upon their caregivers represents a distressing and frequently disregarded facet within the domain of caregiving.

**Objectives:**

This qualitative study aims to 1) explore family caregivers’ experiences of violent, abusive, and harmful behavior by the older person and 2) explore how violent, abusive, and harmful behavior by the older person affects family caregivers’ mental health.

**Methods:**

This qualitative study encompassed 307 participants, with a diverse age range spanning from 65 to 87 years. All the interviews went through the process of content analysis.

**Results:**

For the first objective, findings indicated six emerging themes: Persistent and intense verbal abuse (79.1%); Subjugation and manipulation by older adults (72.5%); Unexpected illicit activities initiated by older adults (62.1%); Financial exploitation by older adults (39.8%); Physical abuse (31.5.%); and Sexual abuse (30.7%). The second objective highlighted four themes: High levels of anxiety and depression (87.9%), Intense rage (79.4%), Feelings of moral isolation (77.4%), and Intense explosions (63.6%). Brazilian participants mainly experienced persistent and intense verbal abuse (64.1%). Moreover, higher levels of depression and anxiety were mainly verbalized by English participants (81.8%).

**Conclusions:**

These findings underscore the significant toll that older individuals’ violent, abusive, and harmful behavior can have on the mental well-being of family caregivers. This study sheds light on the complex experiences faced by family caregivers and emphasizes the urgent need for targeted interventions to foster healthier caregiving environments.

Keywords: Carers; family caregivers; mental health; older adults; violent, abusive and harmful behavior.

**Disclosure of Interest:**

None Declared

